# Primary Repair of Esophageal Perforation Following Anterior Cervical Fusion

**DOI:** 10.7759/cureus.11590

**Published:** 2020-11-20

**Authors:** Fernando Luiz R Dantas, François Dantas, Plínio D Mendes, Bruno L Sandes, Gilberto Fonseca Filho

**Affiliations:** 1 Neurological Surgery, Biocor Instituto, Belo Horizonte, BRA; 2 Neurological Surgery, Hospital Vila da Serra, Belo Horizonte, BRA

**Keywords:** anterior cervical fusion, esophageal perforation, complications, primary closure, primary repair

## Abstract

Introduction

Anterior cervical fusion has been performed safely and effectively for decades for the treatment of multiple pathologies, with low rates of morbidity and mortality. Esophageal perforation is a rare but potentially serious complication of anterior cervical spine approaches. There is no consensus regarding the best treatment strategies for this complication.

Objectives

To determine the prevalence of esophageal perforation following anterior cervical fusion in a single institution and to describe two cases of this complication that were treated with primary repair.

Methods

We retrospectively analyzed all consecutive patients who underwent anterior cervical fusion in a single private institution from January 1999 to August 2017. Patients who developed esophageal perforation per- or postoperatively were included in the analysis.

Results

A total of 830 anterior cervical fusion surgeries were performed during the analyzed period. Two cases (0.24%), both of male patients, were complicated by esophageal perforation, one intraoperatively and the other four years after the first surgery. Both patients were treated with primary esophageal repair, and good outcomes were obtained.

Conclusion

Primary repair is a therapeutic option in cases of esophageal perforation after anterior cervical fusion. Satisfactory results were obtained in both cases. Further studies are necessary to elucidate the best therapeutic options for this rare complication.

## Introduction

Access to the cervical spine through the anterior route has been widely performed since it was described by Smith and Robinson in the late 1950s [1]. The approach is considered safe and is commonly used to treat traumatic, degenerative, and neoplastic diseases. The complication rate of this surgical approach is variable in the literature. Dysphagia is the most frequently found complication, affecting 2% to 67% of patients in some series, and is usually caused by esophageal retraction [2-3].

Esophageal lesions are uncommon, with an estimated prevalence between 0.3% and 4% [4]. They are considered early when they appear before 30 postoperative days and delayed when they are diagnosed after this period [3]. In a series of 17 cases, the presentation was early in 41% and delayed in 59% [5].

In a retrospective study involving 1,015 patients with degenerative cervical spine disease undergoing discectomy and anterior cervical fusion, the mortality rate was 0.1%. In this study, only one death was observed due to accidental esophageal injury [2]. The mortality rate, in general, is about 19% but is related to the period of diagnosis. In lesions diagnosed early, mortality is less than 7% while in cases of late diagnosis, it can reach 27% to 60% [6]. The migration of the plate or screw is the most common cause of late esophageal injury, accounting for 41% of cases [3].

Despite the variable mortality rates reported in the literature, this complication is feared by surgeons due to its difficult therapeutic management.

## Materials and methods

We retrospectively analyzed all consecutive patients who underwent anterior cervical fusion (ACF) surgery at a neurosurgery referral center from January 1999 to August 2017. Data collection was carried out from secondary sources in the medical records. Patients underwent ACF for the treatment of degenerative pathologies, malignancy, disc herniation, or trauma, with standard surgical access on the right. Exclusion criteria were previous surgery performed at another institution and infection. Surgeries were performed by four different neurosurgeons but with similar techniques in all cases. All patients were periodically followed up for a minimum of 24 months, with no loss to follow-up. Patients who presented with intraoperative or postoperative esophageal lesions were included in the analysis.

Our objectives were to determine the prevalence of esophageal perforation following ACF in a private tertiary neurosurgery service and to describe two cases of patients treated with primary repair after the diagnosis of esophageal perforation.

## Results

A total of 830 ACF surgeries were performed during the analyzed period. Only two patients were identified, both male, who presented with esophageal lesions. One patient presented with lesions during the surgery and the other four years after the first surgical approach. Both injuries were treated with primary repair, and a satisfactory response was obtained in the follow-up period without recurrences. The prevalence of esophageal perforation related to ACF surgery in our service was 0.24%. One patient developed pneumonia in the postoperative period, and there was no record of mortality.

Case 1

A 40-year-old male patient, who was a heavy drinker, was a victim of cervical spinal cord trauma with consequent C3-C4 fracture-dislocation. During surgical access to the anterior cervical spine to perform arthrodesis, there was an accidental longitudinal lesion of the esophageal wall at the level of C5-C6 with immediate primary repair performed. The cervical spine procedure was suspended. The patient used a nasoenteric tube for eight days, then resumed an exclusive oral diet, obtaining good evolution and discharge conditions without infectious complications. After 30 days, he was hospitalized again and underwent surgical treatment of the fracture with C3-C4 fusion using plates and screws. The surgical and postoperative periods occurred without abnormalities.

Case 2

**Figure 1:**
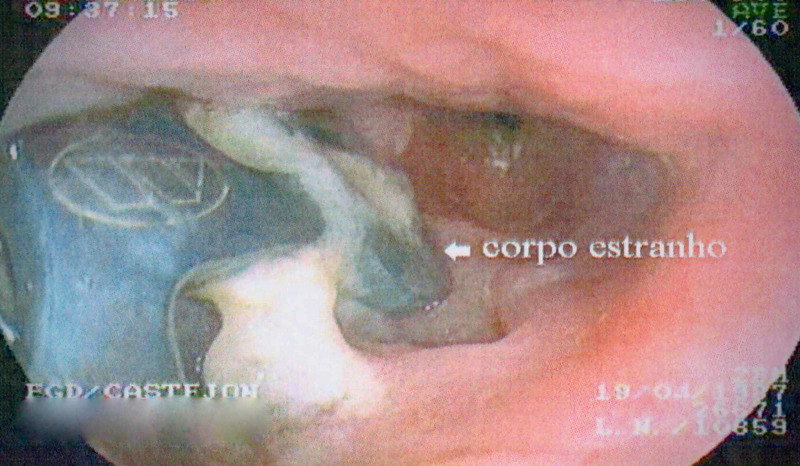
Upper digestive endoscopy showing perforation of the esophagus by the plate used in anterior cervical fusion

**Figure 2:**
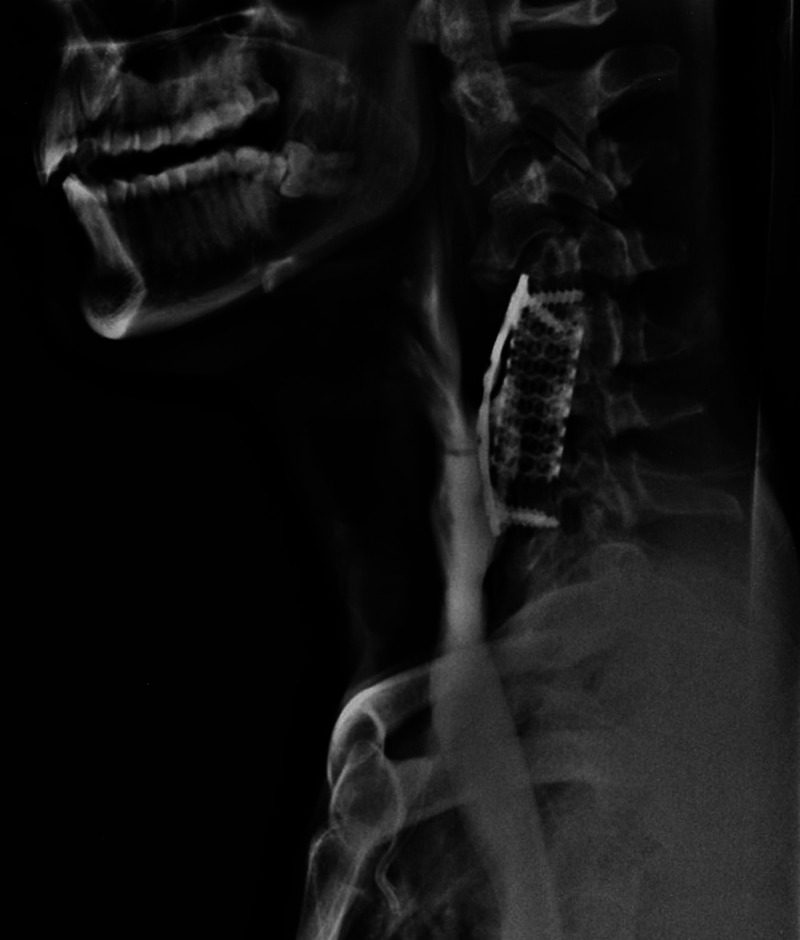
Esophagogram showing esophageal fistula and signs of pseudoarthrosis

**Figure 3:**
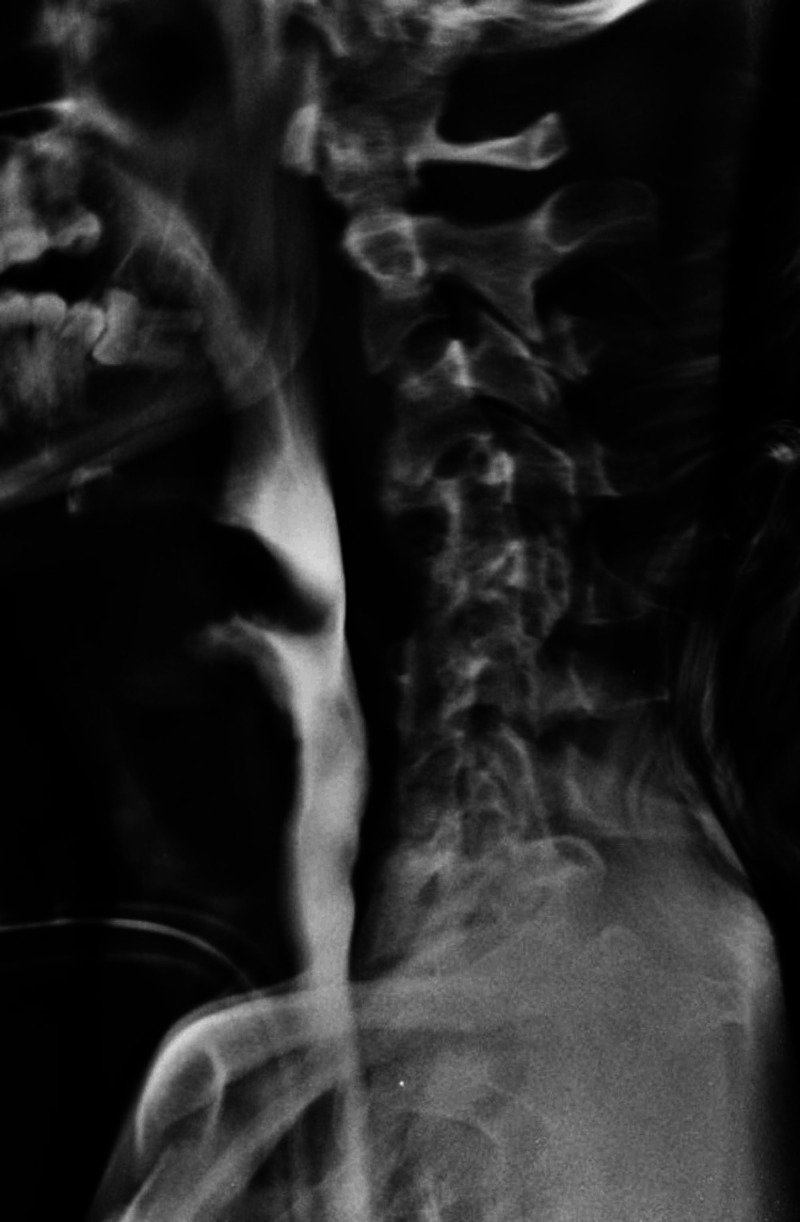
Control esophagogram with no signs of a fistulous path

A 28-year-old male patient was a victim of cervical spinal trauma with complete spinal cord injury after a swimming pool accident four years prior to the development of the esophageal lesion. At the time of the accident, C4-C7 ACF was performed. After one year, reoperation was required due to plate migration. Three years later, he presented to his assistant neurosurgeon due to epigastric pain. Upper gastrointestinal endoscopy showed the perforation of the esophagus by cervical fusion surgical implants (Figure 1). In a complementary study with an esophagogram, an esophageal fistula was found, as well as signs of pseudoarthrosis at the C6-C7 level (Figure 2). Surgical management was performed in conjunction with the general surgery team. The patient underwent plate and cage removal, followed by primary esophageal suturing. In the postoperative period, the patient developed severe pneumonia caused by Acinetobacter baumannii in the intensive care unit, which prolonged his hospitalization for 81 days. Exclusive oral intake was only possible 50 days after surgery. A new esophagogram demonstrated resolution of the fistula (Figure 3). At hospital discharge, he was on an oral diet and had no dyspeptic complaints. After one year, he underwent C2-C6 cervical fusion posteriorly to stabilize the cervical spine.

## Discussion

An esophageal perforation resulting from ACF surgery is an uncommon complication. A recent retrospective study conducted by Hershman et al. found a prevalence of 0.02% in almost 9,600 procedures on the cervical spine via the anterior route [7]. In a systematic review of 240 articles, esophageal injury corresponded to 0.2% of complications of ACF surgery [8]. In our service, the rate of esophageal injury was 0.24%. Despite being an injury feared by every spine surgeon, this complication has a variable rate of morbidity and mortality directly related to the time of diagnosis [7].

The adventitia of the esophagus overlies the outermost esophageal layer, thus protecting the longitudinal and circular muscles underneath, as well as the submucosal and mucous layers. Aggressive or inadequate retraction of the esophagus can result in injury to these layers during cervical surgery. The area of ​​the esophagus most vulnerable to injury, known as Killian's Triangle, is formed by the junction of the paired inferior constrictor pharyngeal and cricopharyngeal muscles. This region, which is usually anterior to the C5-C6 disc but is occasionally found more caudally, is particularly susceptible to injury since the posterior esophageal mucosa lacks muscle protection. At this level, only the thin buccopharyngeal fascia separates the esophagus from the retroesophageal space [7].

The most common lesion location is between levels C5-C6 and C6-C7, and the most common symptoms are dysphagia and odynophagia, followed by fever and cervical edema. The clinical presentation may include persistent dysphagia, which may be isolated or accompanied by other signs and symptoms such as fever, increased wound volume, and skin fistula [3]. The classic triad of vomiting, chest pain, and subcutaneous emphysema, known as Mackler's triad, is seen in only 25% of cases and is more common in thoracic esophageal lesions [7,9].

In cases of associated infection, the most commonly found pathogens are Staphylococcus and Streptococcus, followed by Pseudomonas, Klebsiella, Candida, Acinetobacter, and Lactobacillus [8].

Another interesting feature of esophageal lesions in ACF is the time of diagnosis. On average, the diagnosis of esophageal perforation occurs two years after surgery [3], which is generally longer than the usual follow-up time for these patients after the procedure. There are reports of very late clinical manifestations, including a diagnosis that was registered 18 years after the surgical procedure [10]. Therefore, it is important to advise patients on possible late complications involving cervical spine surgery via the anterior route [3].

The key to the immediate treatment of esophageal perforation is an accurate diagnosis and assessment of whether surgery is necessary. Brinster et al. proposed a treatment algorithm but did not specify whether the injury would be cervical, thoracic, or abdominal, and did not take into account the arthrodesis implants [11].

There is a divergent opinion regarding the best diagnostic evaluation between contrast esophagogram versus computed tomography (CT) or magnetic resonance imaging (MRI) as the primary diagnostic modality for esophageal perforations. The esophagogram determines the location, size of the perforation, and the presence of a pseudo-diverticulum or associated mediastinitis [6]. A contrasted CT scan of the neck has a sensitivity of 92% to 100% [12]. The contrasted esophagogram has a false negative rate of around 25%, especially if it is done in the acute phase, due to the edema associated with a small perforation [6-7], with a sensitivity of 50% to 89% [6,11,13]. According to some authors, esophagography and contrasted CT of the esophagus should always be performed [14]. The maximum number of examinations necessary should be performed before a surgical approach is indicated.

Intraoperative lesions of the esophageal wall represented 19% of the cases in a systematic review of 153 patients by Halani et al. [3]. Late esophageal perforation is a rare complication of ACF. The most frequent cause of late presentation is hardware failure, that is, migration of the plate, screws, or both (41%), followed by chronic erosion by hardware (31%), intraoperative injury (19%), and graft extrusion and penetration (7%) [3]. Nakano et al. recently conducted an interesting study on the use of intraoperative CT to prevent possible iatrogenic esophageal injuries. The authors demonstrated that, at the level of C6 or at the most caudal levels, there is a greater possibility of intraoperative esophageal injury caused by retractors [15].

In our series, one of the cases was diagnosed intraoperatively, and another case was diagnosed four years after the first surgery with esophageal perforation by the synthesis material. These cases were at the C5-C6 and C6-C7 levels, respectively.

Recently, a case was described in which erosion against the pharyngeal mucosa culminated in the complete extrusion of the fixation construct orally three and a half years after C2-C3 arthrodesis [16]. A similar case was reported by Sharma et al. in 2001, with late oral graft extrusion after C2-C3 arthrodesis [17]. Pompili et al. described a case of asymptomatic esophageal perforation by an extruded screw, with probable expulsion through the intestinal tract [18]. However, reports of esophageal erosion after ACF are not only related to modern synthetic materials. In a case report published by Raso et al. in 1985, the authors described the oral expulsion of an acrylic prosthesis implanted in a patient [19].

The treatment of esophageal perforation after fusion is complex and without consensus [3-4,7]. Most studies are case reports or small case series [6,10,14,20-25]. Conservative management is usually indicated in young patients with lesions smaller than 1 cm, who are asymptomatic and without infectious signs [4]. Surgical treatment, however, is recommended for patients with suspected infection, fistula, or the presence of a pseudocyst identified by contrast tests [6].

Repair with primary esophageal raffia is a therapeutic option, although there are reports of a higher rate of failure and stenosis with this procedure [4]. Another treatment option is the use of a pedicled muscle, primarily the sternocleidomastoid, to reinforce the repair. Other options include the forearm muscles, pectoralis major muscle, omohyoid, great dorsal, longus colli, omentum, or jejunum [3-4,6,20,26]. In addition to being used as a physical barrier, the use of a pedicled muscle provides greater antibiotic distribution due to the greater vascular supply generated [6].

Ghirelli et al., in a series of 17 cases in which all patients underwent direct suture (most of them with the interposition of a pedicled flap), reported good results with return of oral intake in 16/17 cases; one patient had a severe neurological condition. The return of oral intake ranged from nine days to 383 days (average of 68 days). In early cases, the average was 55 days, better when compared to 87 days in delayed cases [5]. In our series, exclusive oral intake was possible within eight days in the first case and 50 days in the second case. In a systematic review by Halani et al., there was no difference in the time to the beginning of oral intake between primary repair or the use of a muscle flap (28.3 and 27.3 days, respectively) [3].

Studies indicate that the etiology of the esophageal lesion can determine the best therapeutic option. In intraoperative lesions, primary repair is the option of choice, as the damage is generally small, while the lesions diagnosed late tend to be larger and, therefore, would benefit from reinforcement with a pedicled muscle [3,5,7].

The rate of postoperative complications after repair is high in the literature, ranging from 75% to 86%, with pneumonia being the most common [3,5].

There is no consensus in the literature regarding the need for posterior fixation in cases of pseudoarthrosis. In the series by Ghirelli et al., 10/17 patients underwent posterior cervical fusion before correction. In all cases where materials such as plates and cages were present, the implants were removed [5].

In the cases we described, we opted for isolated primary repair and obtained a satisfactory result in follow-up for 18 years in the first patient and for four years in the second. With regard to late esophageal perforation, we consider that patients diagnosed years after fusion can benefit from primary repair since the denser fibrotic scar tissue can provide better quality sutures.

## Conclusions

Esophageal perforation after anterior cervical fusion is a rare and dangerous complication. The most common causes are erosion in the esophagus caused by the plate and migration of the synthesis material. Patients with persistent dysphagia after ACF should be evaluated for this possibility. In addition, they should be advised about the warning signs, which may appear even years after the surgery. Therapeutic management is challenging, with primary repair being an effective option for specific cases. Treatment must be multidisciplinary, including neurosurgery, general surgery, general practice, and nutrition. Although this condition is often serious and may be fatal, early diagnosis and prompt therapeutic intervention, when indicated, represent the essential pillars for a favorable outcome.
